# Structure and function of chicken interleukin-1 beta mutants: uncoupling of receptor binding and *in vivo* biological activity

**DOI:** 10.1038/srep27729

**Published:** 2016-06-09

**Authors:** Wen-Ting Chen, Wen-Yang Huang, Ting Chen, Emmanuel Oluwatobi Salawu, Dongli Wang, Yi-Zong Lee, Yuan-Yu Chang, Lee-Wei Yang, Shih-Che Sue, Xinquan Wang, Hsien-Sheng Yin

**Affiliations:** 1 Institute of Bioinformatics and Structural Biology, and College of Life Sciences, National Tsing Hua University, No. 101, Section 2, Kuang-Fu Road, Hsinchu 30013, Taiwan; 2 Bioinformatics Program, Taiwan International Graduate Program, Academia Sinica, Taipei, 115, Taiwan; 3 School of Life Science, Tsing Hua University, Beijing, China

## Abstract

Receptor-binding and subsequent signal-activation of interleukin-1 beta (IL-1β) are essential to immune and proinflammatory responses. We mutated 12 residues to identify sites important for biological activity and/or receptor binding. Four of these mutants with mutations in loop 9 (T117A, E118K, E118A, E118R) displayed significantly reduced biological activity. Neither T117A nor E118K mutants substantially affected receptor binding, whereas both mutants lack the IL-1β signaling *in vitro* but can antagonize wild-type (WT) IL-1β. Crystal structures of T117A, E118A, and E118K revealed that the secondary structure or surface charge of loop 9 is dramatically altered compared with that of wild-type chicken IL-1β. Molecular dynamics simulations of IL-1β bound to its receptor (IL-1RI) and receptor accessory protein (IL-1RAcP) revealed that loop 9 lies in a pocket that is formed at the IL-1RI/IL-1RAcP interface. This pocket is also observed in the human ternary structure. The conformations of above mutants in loop 9 may disrupt structural packing and therefore the stability in a chicken IL-1β/IL-1RI/IL-1RAcP signaling complex. We identify the hot spots in IL-1β that are essential to immune responses and elucidate a mechanism by which IL-1β activity can be inhibited. These findings should aid in the development of new therapeutics that neutralize IL-1 activity.

Interleukin-1 beta (IL-1β) plays a central role in coordinating host immune and proinflammatory responses, and it has been shown to enhance production of immune-related molecules, e.g., adrenocorticotropin, cytokines, and chemokines^1^,^2^,^3^,^4^. IL-1β is produced by macrophages and monocytes and is synthesized as a propeptide^5^. The mature, bioactive form is produced upon caspase 1–mediated proteolysis of the pro-IL-1β. Interaction of IL-1β with the type I IL-1 receptor (IL-1RI) elicits a cascade of immune responses^5^. IL-1β has been used as a vaccine adjuvant to enhance the immune response against pathogens such as influenza virus^6^, *S. pneumonia*^7^, and coccidiosis^8^.

An increasing number of reports have characterized the structure and function of avian IL-1βs^9^,^10^,^11^,^12^. The sequence identities for human and avian IL-1βs are only 31 to 35%^10^,^13^. Three-dimensional structures of chicken and human IL-1βs show a similar structural fold, i.e., they contain one or two α-helices and 12 to 14 β-strands, which form an antiparallel β-barrel, with a shallow open face at one end and a closed face at the other^11^,^14^. However, human IL-1β cannot induce chemokine expression in chicken fibroblasts or elevate the plasma cortisol level in chickens, thereby demonstrating a lack of cross-species bioactivity^11^,^15^. Close examination of the chicken and human IL-1βs tertiary structures indicates that major differences are found for loops 3, 4, 7, 8, and 9. The residues located in these loops may be critically important for receptor binding, accounting for the differences in cross reactivities and immunological responses^11^,^13^.

The structure of human IL-1β in complex with its type I interleukin-1 receptor (IL-1RI) has been determined^16^. A large-scale mutagenesis study revealed that human IL-1β binds to its IL-1RI at two sites, labeled IL-1β A and B^16^. Site A contacts domains 1 and 2 of IL-1RI and sites B contacts domain 3 of IL-1RI. The crucial residues of human IL-1β involved in receptor-mediated biological activity or receptor binding have been identified by site-directed mutagenesis^17^,^18^,^19^,^20^. R4, Q15, Q32, and K93 in human IL-1β interact with the receptor via intermolecular hydrogen bonds. L10, R11, H30, F46, I56, K103, and E105 are also involved in receptor binding. In contrast to human IL-1β, the residues in chicken IL-1β important for biological activity or receptor binding have not been identified. Recently, a structural model of a chicken IL-1β/chicken IL-1RI complex was built^11^,^13^. Four chicken IL-1β residues, R8, E25, R52, and R54, are within hydrogen-bonding distance of receptor residues and can be positioned to form salt bridge with the receptor in the model. In addition, 10 residues, T7, N18, Q19, H34, Q36, S39, S40, Q64, T117, and Q138 can be positioned to form hydrogen bonds with the receptor in the model. These residues are shown in black and along with other receptor-binding residues highlighted in gray in Fig. 1.

Herein, we report the results of an extensive site-directed mutagenesis study aimed at identifying residues in chicken IL-1β responsible for biological activity as defined by an induced *in vivo* increase of chicken plasma cortisol. Additionally, circular dichroism (CD) spectroscopy, surface plasmon resonance experiments, and X-ray crystallography were used to characterize the physiological significant receptor-binding affinity, and structural basis of some of the chicken IL-1β mutants found to decrease *in vivo* activity. *In silico* docking and molecular dynamics simulations were used to explore the structural properties and differences of some of the chicken IL-1β mutants with decreased *in vivo* activity. This study expands our understanding of residues important to IL-1β biological activity, receptor binding, and the immune response.

## Results

### Design of chicken IL-1β mutants

**Figure 1:**
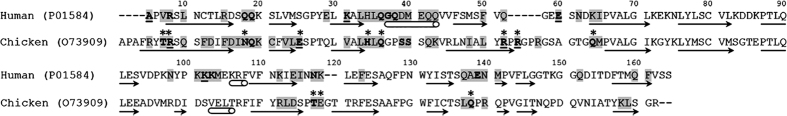
Multiple sequence alignment of human and avian IL-1βs. Sequences used in the alignment are those of human IL-1β (UniProtKB:P01584) and chicken IL-1β (UniProtKB:O73909). Accession numbers are given in the parentheses. Residues directly involved in receptor binding are shaded in gray. Residues that form hydrogen bonds or salt bridges with receptor residues are in bold type or underlined, respectively^13^. The stars indicate the chicken IL-1β residues selected for in this study.

We previously performed molecular dynamics (MD) simulations for the chicken complex of IL-1β/IL-1RI where the avian IL-1β structure was solved by us and the IL-1RI was homology-modeled from its human equivalent (PDB: 4DEP)^13^. MD-refined complex structure revealed the IL-1β/IL-1RI interface^13^ and the residues in chicken IL-1β that made broad contacts with receptor IL-1RI via intermolecular hydrogen bonds were selected for alanine-scanning mutagenesis. Genes for the chicken IL-1β mutants T7A, R8A, N18A, E25A, H34A, Q36A, R52A, R54A Q64A, T117A, E118A, E118K, E118R, and Q138A were designed and then prepared by site-directed mutagenesis. The genes were expressed in *E. coli* BL21 (DE3) and purified to homogeneity by Co^2+^-affinity chromatography using their histidine tags (Supplementary Experimental procedures, Supplementary Table 1, and Supplementary Fig. 1). Most of the mutated residues are directly involved in receptor binding via hydrogen-bonds or salt bridges (Fig. 1). The residues near the N-terminus, i.e., T7 and R8, are found in the first β strand of IL-1β. R8 is conserved in chicken, human, and mouse IL-1βs and is important for receptor binding^20^. Residue N18 is part of loop 1, which is between the first and second β strand. E25 is found in the second β strand of IL-1β; H34 and Q36 are located in loop 3, which is between the third and fourth β strand, and are conserved in human and chicken IL-1β. The corresponding residues play a crucial role in the binding of human IL-1 β to its receptor^17^,^19^. By examining the human and chicken IL-1β structures and sequences, we noted that loop 4, which contains R52, R54, and Q64 and is between fourth and the fifth β strand, and loop 9, which contains T117 and E118, and is between ninth and the tenth β strand, are conformationally different in comparison with the corresponding loops in human IL-1β^13^. Q138 in loop 11, near the C-terminus, is part of site A.

### *In vivo* biological activity of IL-1β mutants

**Figure 2:**
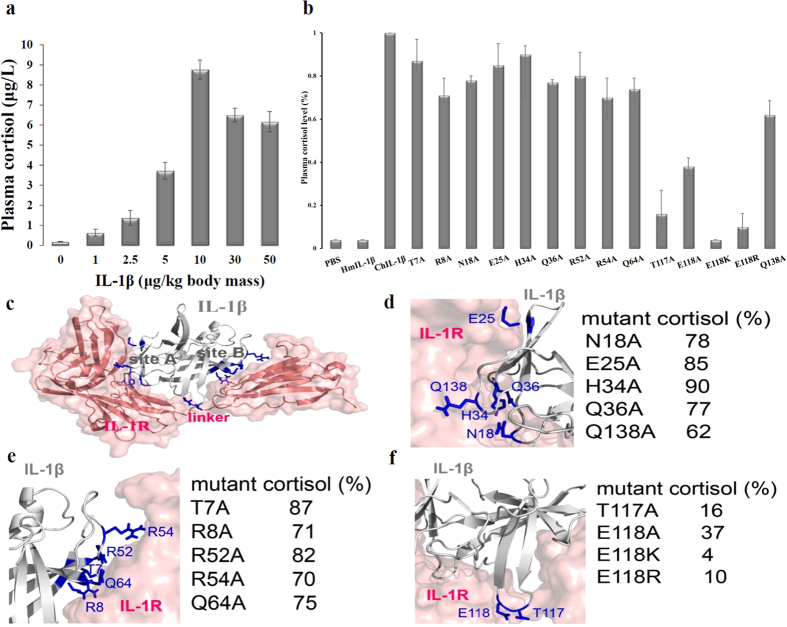
*In vivo* functional assay of IL-1β mutants. (**a**) Cortisol production is induced in a dose-dependent manner by chicken IL-1β and released into the plasma. (**b**) To determine the bioactivities of WT chicken IL-1β (ChIL-1β), T7A, R8A, N18A, E25A, H34A, Q36A, R52A, R54A, Q64A, T117A, E118A, E118K, E118R, and Q138A, and human IL-1β (HmIL-1β), each protein was directly and individually injected into a wing vein of three adult chickens, after which the plasma cortisol levels were measured. (**c**) Ribbon diagram of chicken IL-1β (grey) complexed to IL-1R (pink). Mutation sites are shown in blue. Mutated residues in site A, site B, and loop 9 are shown in (**d**–**f**), respectively with the cortisol levels relative to that of WT IL-1β shown to the right of the mutant names.

To test the linearity of the *in vivo* IL-1β bioassay, various concentrations of WT chicken IL-1βs were injected into a wing vein of adult chickens and changes in plasma cortisol levels were measured (Fig. 2a). The concentration of cortisol in plasma increased from a basal level of 0.18 μg/L (obtained from the injection of 0 μg protein/kg body mass) to the maximal level of 8.77 μg/L (obtained from the injection of 10 μg protein/kg body mass). The effects of IL-1β on cortisol induction were concentration dependent. Thus, measurement of corticosterone in chicken serum, post an intravenous injection (10 μg protein/kg body mass) of recombinant chicken IL-1β into adult chicken, was chosen and used as an indication of biological activity for chicken IL-1β *in vivo*. The delivery significantly elevated plasma cortisol that reached its maximal level at 1 h post injection^9^. To compare the effects of the IL-1β mutants on the chicken immune system, we individually injected purified WT human, WT chicken, and mutant chicken IL-1βs directly into wing veins of adult chickens, and then measured the plasma cortisol levels at 1 h (Fig. 2b). Among the mutants tested, T117A and E118A did not increase the plasma cortisol levels to the extent that WT chicken IL-1β did (*p* < 0.05) (Fig. 2b). To further assess the biological role of E118, we prepared E118K and E118R with positively charged side-chains replacing the negative side chain of glutamate. The plasma cortisol concentrations remained at near basal levels after injecting E118K and E118R (Fig. 2b,f). The mutants with replacements in loops 1 (N18A), 3 (H34A and Q36A), 4 (R52A, R54A, and Q64A), 11 (Q138A), and in β strands 1 (T7A and R8A) and 2 (Q25A) caused an increase in plasma cortisol expression similar to as that of WT IL-1β, whereas injection of phosphate-buffered saline or human IL-1β purified from the same protein purification method^11^ did not increase the plasma cortisol concentration (Fig. 2b). It indicated that the increased or decreased cortisol concentration in the serum resulted from responses to a specific stimulus chicken IL-1β (or its mutants). The difference between the plasma cortisol values induced by WT chicken IL-1β and phosphate-buffered saline were statistically significant (*p* < 0.05).

Upon examination, we noted that mutations of IL-1 β residues in site A caused a minor to moderate loss of biologic activity (Fig. 2d), with the induced cortisol levels relative to that of WT chicken IL-1β being 78%, 85%, 90%, 77%, and 62% for N18A, E25A, H34A, Q36A, and Q138A respectively. Mutations in site B also induced little loss in biologic activity (Fig. 2e), with the cortisol levels relative to that of WT chicken IL-1β being 87%, 71%, 82%, 70% and 75% for T7A, R8A, R52A, R54A, and Q64A, respectively. Conversely, large decreases in activity were found for mutations in loop 9 (Fig. 2f), The induced cortisol levels relative to WT IL-1β are 16%, 37%, 4%, and 10% for T117A, E118A, E118K, and E118R, respectively, indicating that these proteins should be able to substantially block *in vivo* chicken pro-inflammatory immune responses.

### Far-UV CD spectra of T117A, E118A, E118K, E118R, and WT IL-1β

**Figure 3:**
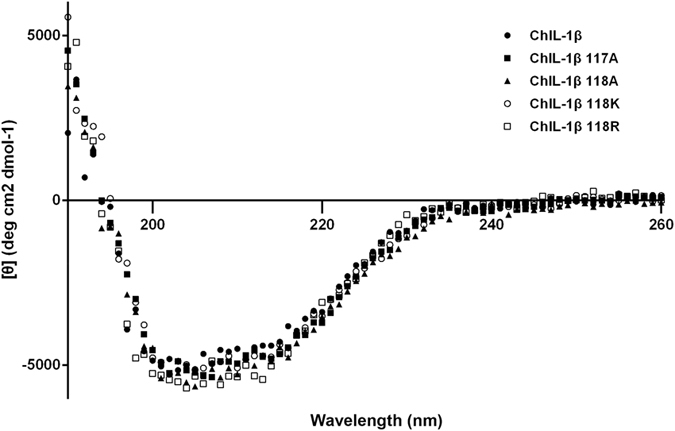
Far-UV CD spectra of WT chicken IL-1 β, T117A, E118A, E118K, and E118R.

To determine if the mutations in T117A, E118A, E118K, and/or E118R would substantially disrupt their tertiary structures and would thereby cause reduction in biological activity, the secondary structures of WT IL-β and the four aforementioned mutants, were assessed by far-UV CD spectroscopy. The five spectra are nearly identical and have minima at ~206 nm (Fig. 3), which indicates the presence of β-sheet.

### Effects of 117A, and 118K on receptor binding

**Figure 4:**
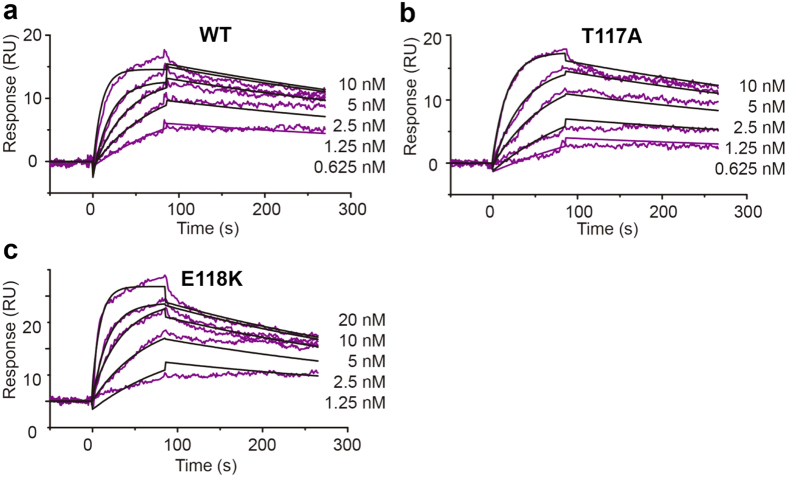
Surface plasmon resonance assays to determine the binding affinities of WT chicken IL-1β and its mutants to chicken IL-1RI. (**a**) WT chicken IL-1β, (**b**) T117A, and (**c**) E118K. Sensorgrams were obtained by injecting various concentrations of WT IL-1β or a mutant over a chip with surface-immobilized IL-1RI. The experimental data (red lines) were fit with a 1:1 interaction model (black lines). RU, response unit.

To assess if the significantly reduced biological activities of T117A, E118A, and E118K was a consequence of reduced receptor-binding activity, a surface plasmon resonance study was performed using immobilized IL-1RI and a free mutant or WT chicken IL-1β (Fig. 4). The K_d_ values for WT IL-1β, T117A, and E118K were 0.12, 0.23, and 0.37 nM, respectively, suggesting that that three mutants have similar receptor binding affinities compared with WT chicken IL-1β. Thus, the mutated residues in loop 9 seem to have no great effect on receptor binding even though they significantly reduce biological activity, which suggests that these residues may be involved in signal transduction once IL-1β has bound to the receptor.

### Effects of 117A, and 118K on IL-1β signaling *in vitro*

**Figure 5:**
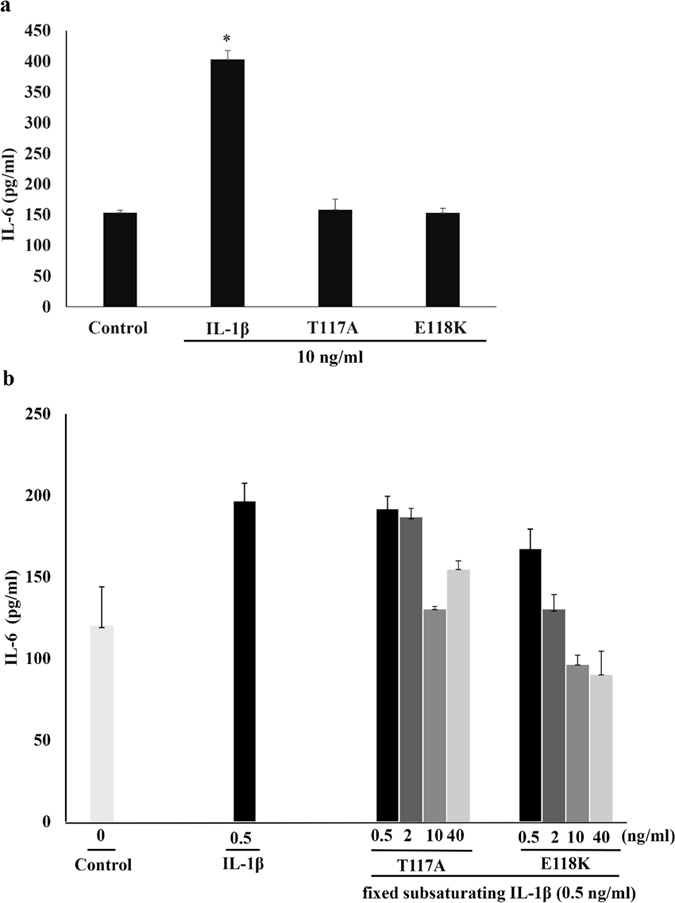
(**a**) Effects of chicken WT IL-1β, T117A, and E118K on IL-6 production in DF-1 chicken fibroblast cell line. DF-1 cells were treated with DMEM (Control) or 10 ng/ml of chicken WT IL-1β, T117A, and E118K for 8 h. (**b**) Inhibition of chicken WT IL-1β activity by T117A and E118K mutants. DF-1 cells were treated with increasing concentration (0–40 ng/ml) of mutants T117A or E118K for 8 h in the presence of fixed sub-saturating concentration (0.5 ng/ml) of WT IL-1β. Those cell lysates were collected and measured for IL-6 production using chicken-specific ELISA. Data are shown as the mean ± SD of triplicate determinations *p < 0.05, IL-1β vs control.

To evaluate the effects of T117A and E118K on IL-1β signaling *in vitro*, IL-6 production induced by chicken IL-1β was evaluated by measuring the IL-6 expression level in chicken fibroblast cell lysates (Fig. 5a). The results showed that 10 ng/ml of WT chicken IL-1β induced IL-6 synthesis in the DF-1 chicken fibroblast cells, while the treatment of T117A or E118K showed no effect in increasing the IL-6 expression. The elevation of IL-6 expression induced by chicken IL-1β is statistically higher (*p* < 0.05) than that by control, T117A and E118K, while the two mutants had a statistically indistinguishable effect on IL-6 production from that of the control. In addition, to determine whether mutants T117A and E118K antagonize chicken IL-1β activity, we examined how much IL-1β induced IL-6 expression in DF-1 cells in the presence of mutants. Interestingly, these mutants of chicken IL-1β exhibited dose-dependent inhibition of IL-1β-induced IL-6 production to act as antagonists (Fig. 5b).

### Crystal structures of T117A, E118A, and E118K

A total of 154, 149, and 159 residues and 47, 27, 43 water molecules were resolved in the crystal structures of T117A, E118A, and E118K, respectively. No electron densities were found for residues 1, 2, 55–59, and 162 in T117A; residues 1, 2, 37–40, 56–61, and 162 in E118A; and residues 1, 2, and 162 in E118K. In addition, no electron density was found for the His_6_-T7 tags (34 residues) in the three mutants. We used PROCHECK^21^ to examine the stereochemical quality of the final structures. All stereochemical parameters for side-chain and main-chain atoms were within acceptable limits, with the ϕ-ψ values of the residues being in the most favored or allowed regions of the Ramachandran plots (Supplementary Table 2).

**Figure 6:**
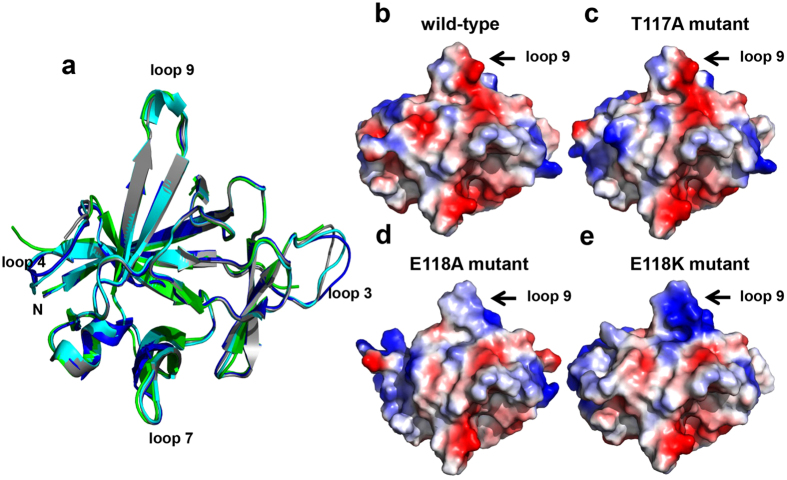
Chicken WT IL-1β, T117A, E118A, and E118K structures. (**a**) Superimposed ribbon diagrams of IL-1β, T117A, E118A, and E118K. (**b**–**e**) The electrostatic potential surfaces were generated by PyMOL. Positively charged regions are shown in blue, and negatively charged regions are in red. Arrows identify loop 9 where differences between the electrostatic potentials for WT IL-1β and its mutants were found.

The crystal structures of the mutants contain the same β-strands and the α-helix found in WT IL-1β, and the β-strands exhibit a typical beta-trefoil fold nearly identical to that of WT IL-1β^11^ (Fig. 6a). Individual superimposition of each mutant structure onto that of IL-1β shows that the structures share a very similar fold, i.e., rmsd values for IL-1β compared with T117A, E118A, and E118K are 0.518 Å, 0.783 Å, and 0.403 Å, respectively. Close examination revealed a dramatic conformational change for loop 9 in WT IL-1β and E118K involving residues 116–118, i.e., these residues form a loop in WT IL-1β, but a short α-helix in E118K (Fig. 6a).

Our *in vivo* immune-response bioassays revealed significant differences in the activities for WT IL-1β, T117A, E118A, E118K, and E118R as noted above. Using our crystal structures we generated electrostatic potential maps for WT IL-1β and the three mutants (Fig. 6b–e). We found that the charge distributions near loop 9 differ for WT IL-1β and E118A and E118K. The loop 9 region is predominantly negatively charged in WT IL-1β (Fig. 6b), whereas that region in T117A (Fig. 6c) and E118A (Fig. 6d) have reduced negative charges; the loop 9 region in E118K has a relatively greater area of positive charge than does WT IL-1β (Fig. 6e).

### Mutations in IL-1β loop 9 impair the complex stability, revealed by crystal structures and potential energy calculations

**Figure 7:**
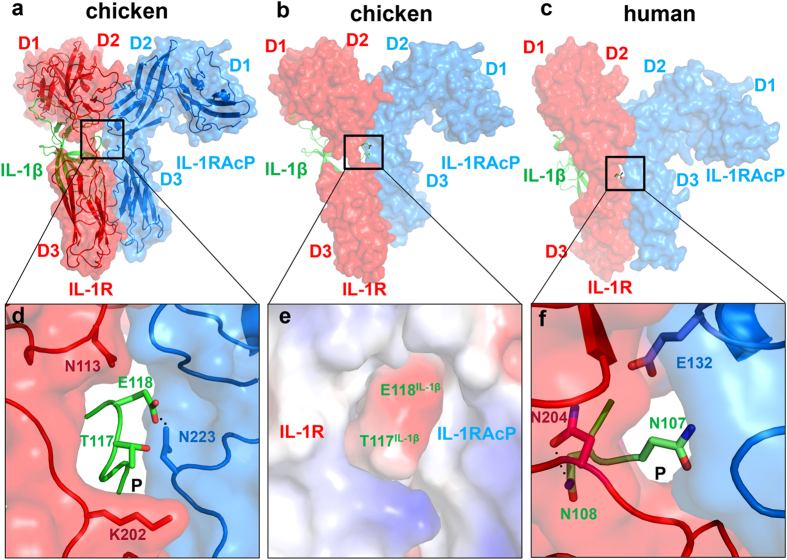
Structural models of human and chicken IL-1β/IL-1RI/IL-1RAcP complexes. IL-1β is shown as a green ribbon diagram in panels (a–c). IL-1RI and IL-1RAcP is shown in red and blue ribbon and/or surface diagrams, respectively. (**d**) A hydrogen bond is formed between the side chains of IL-1β E118 and IL-1RAcP N223 in the interface pocket (P). (**e**) Electrostatic potential surface surrounding chicken IL-1β T117 and E118 at the chicken IL-1RI/IL-1RAcP interface. Positively and negatively charged surfaces are shown blue and red, respectively. (**f**) Interactions surrounding human IL-1β residues N107 (T117 in chicken IL-1β) and N108 (E118 in chicken IL-1β) at the human IL-1RI/IL-1RAcP interface (PDB: 4DEP).

As described in the material and methods, chicken IL-1β/IL-1RI/IL-1RAcP complex structure is modeled from its human equivalent (PDB: 4DEP). In the context of the ternary complex, it can be seen that IL-1β loop 9 sticks into a crevice between IL-1RI and IL-1RAcP (Fig. 7). The loop 9 interacts with chicken IL-1RAcP and the IL-1RI linker via hydrogen bonds and hydrophobic interactions, respectively (Fig. 7a,d). Specifically, chicken IL-1β T117 interacts with IL-1RAcP N223 and IL-1RI K202, and IL-1β E118 forms a hydrogen bond with IL-1RAcP N223 with its backbone interacting with IL-1RI N113 (Fig. 7d). This configuration is also observed in human IL-1RI/IL-1RAcP (Fig. 7c,f). Human IL-1β N108 (T117 in chicken IL-1β) hydrogen bonds to IL-1RI N204 (P200 in chicken IL-1RI) and electrostatic interactions are formed between IL-1β N107 (E118 in chicken IL-1β) and IL1RAcP E132 (Fig. 7f). In general, the electrostatic potential surface involving residues T117 and E118 are more negatively charged than its surroundings, confined by IL-1RI/IL-1RAcP (Fig. 7e). Potential energy calculations based on GROMOS96 45A3 force field show that the loop 9 –IL-1RI/IL-1RAcP interactions are energetically less favorable for the mutant complexes such that mutants T117A, E118A, E118K, and E118R resulted in an increased energy, as compared to the WT, by 177.1, 498.6, 54.1, and 72.6 kJ/mol, respectively, which could destabilize the chicken IL-1β/IL-1RI/IL-1RAcP complex.

### MD simulations

**Figure 8:**
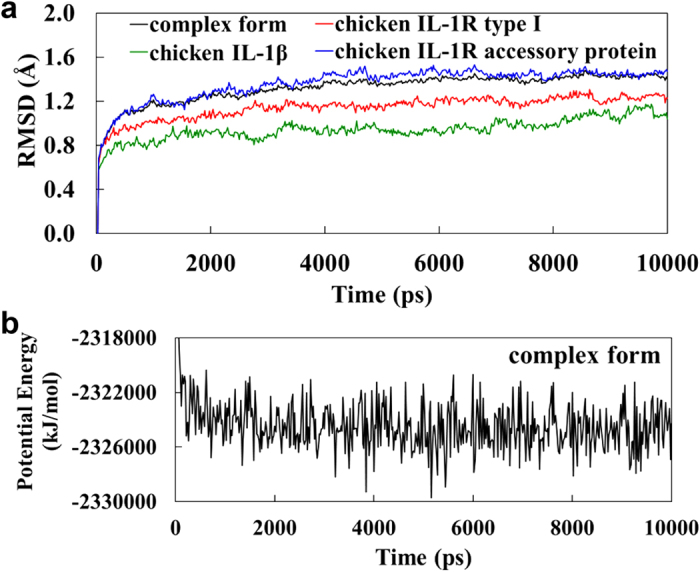
MD trajectories for free chicken IL-1β, IL-1RI, IL-1RAcP and the IL-1β/IL-1RI/IL-1RAcP complex. (**a**) Rmsd values (from the first frame of the NPT runs) for the heavy atoms in chicken IL-1β (green line), IL-1RI (red line), and IL-1RAcP (blue line) and the ternary complex (black line) during the MD run. (**b**) Potential energy for IL-1β/IL-1RI/IL-1RAcP was plotted as a function of time.

To assess the quality of the MD simulations, we examined the rmsd values of all heavy atoms during the IL-1β, IL-1RI, IL-1RAcP, and IL-1β/IL-1RI/IL-1RAcP runs (Fig. 8a). The complex and its constituent monomers attained their equilibrium structures at ~4.5 ns and remained stable until the 10-ns ends of the trajectories. Overall, the IL-1β simulation had the least structural variation, followed by that of IL-1RI. The IL-1RAcP simulation had the greatest degree of structural variation. The potential energy of the complex during and at the end of the simulation indicated that the system was stable and at equilibrium over the course of the run (Fig. 8b).

## Discussion

The biological activity of the chicken IL-1β mutants was assessed by measuring the plasma cortisol level in chicken. Among the fourteen mutants tested, the three that had a mutation in loop 9 (residues 116–118) showed a significant loss of biological activity, but could still effectively bind chicken IL-1RI, indicating that loop 9 is involved in bioactivity but not in receptor binding.

In this study, three types of mutant chicken IL-1βs were designed and expressed that had a single substitution in the N-terminal region or in a loop that might be involved in receptor binding (Fig. 2). The first type involved substitution of alanine for N18, E25, H34, Q36, or Q138 as these residues are part of binding site A of chicken IL-1β. Substitution of these residues resulted in a loss of 10–40% of the biological activity as defined by an increase in the plasma cortisol level in mice relative to that of WT IL-1β. The second type involved substitution of alanine for T7, R8, R52, R54, or Q64 as these residues are located in binding site B of chicken IL-1β. Only a slight reduction in biological activity was observed for the corresponding mutants.

Interestingly, four mutants, T117A, E118A, E118K, and E118R with mutations in loop 9, of chicken IL-1β have no significant effect on receptor binding but significantly reduce biological activity, which suggests that loop 9 is involved in downstream signal transduction. To characterize whether T117A and E118K stimulate IL-1 signaling pathway *in vitro*, IL-6, the key inflammatory mediator triggered by IL-1β through activation of p38 and NFκB signaling pathway, was selected to assess the IL-6 synthesis effect^22^,^23^,^24^,^25^. Chicken WT IL-1β induces significant synthesis of IL-6 and the synthesis majorly occurs in the intracellular compartment. The effect is similar to that of human orbital fibroblast^23^. However, T117A and E118K fail to enhance the IL-6 synthesis. It indicates that the two mutations T117A and E118K lack the ability to active the IL-1 signaling *in vitro*. Treatment of T117A or E118K in DF-1 cells still mildly induces the same level of IL-6 as DF-1 cell alone. It implies that T117A and E118K cannot induce secretion of endogenous IL-1β that exists in chicken fibroblast cells. Interestingly, T117A and E118K lost the biological activity but maintained binding to IL-1RI. They thus serve antagonists and inhibit WT IL-1β-induced IL-6 production competitively. These mutants may become helpful tools for the investigation of chicken IL-1 signal transduction mechanisms. In addition, CD spectroscopy of these mutants showed that they have the same secondary structure content as WT IL-1β, i.e., they are mostly β-sheet in nature, suggesting that the mutants are properly folded. Our crystallography data indicates that the three mutants and WT IL-1β have the same space group as native IL-1β, suggesting that their molecular packing is unrelated to their biological function or receptor binding. We also characterized the structural and functional roles of loop 9 residues T117 and E118 using *in silico* docking and molecular dynamics of IL-1RI, WT IL-1β, T117A, E118A, and E118K as free molecules and in their binary complexes (Supplementary Fig. 2). Loop 9 in WT IL-1β and these mutants closely contacts the linker in IL-1RI that connects domains 2 and 3. Notably the loop 9 residues are extended in E118A (Supplementary Fig. 3c) and form a short helix in E118K (Supplementary Fig. 3d). Loop 9 of human IL-1β also interacts with the domain 2/3 linker in IL-1RI, and this linker helps maintain the domain 3 configuration that appears to be required for signal transduction^26^. Possibly, the different structural features of loop 9 in human and chicken IL-1β are crucial for receptor binding such that the IL-1βs from the two species do not cross react. The region surrounding loop 9 is predominantly negatively charged in WT chicken IL-1β but has the opposite charge in human IL-1β. E118A and E118K also have a charge distribution that is opposite WT chicken IL-1β (Fig. 6d,e) and altered structures (Fig. 6a) in loop 9. Our results suggest that loop 9 in chicken IL-1β may be involved in IL-1β signal transduction.

Signal transduction of human IL-1β is initiated by forming a heterotrimeric complex involving the ligand IL-1β, receptor IL-1RI, and receptor accessory protein IL-1RAcP. Recently, the crystal structure of human IL-1β in complex with the ectodomains of IL-1RI and the IL-1RAcP was determined and showed that the architecture of IL-1β/IL-1RI is nearly unchanged when IL-1RAcP binds^27^. To gain insight into the mechanism by which chicken IL-1β binds to its receptor and receptor accessory protein, 3D structural models for chicken IL-1RI and IL-1RAcP were constructed by homology modeling of the chicken sequence based on known structures of human IL-1RI and IL-1RAcP. We used this structure as the template for our *in silico* docking and molecular dynamics simulations so as to examine the interface around IL-1β loop 9. By replacing E117 with an alanine or lysine and E118 with an alanine, the resulting mutants would not be able to form the wild-type hydrogen bond and hydrophobic interaction[s], and would present an unfavorable charge to the IL-1RI/IL-1RAcP interface. The conformation of loop 9 in T117A, E118A, and E118K may disrupt structure packing and protein stability required for IL-1β signaling after complexation.

In summary, our crystallographic data, *in silico* simulations, and biological results identify T117 and E118 as “hot spots” in the IL-1β immune response and help elucidate a mechanism by which IL-1 activity can be inhibited. These findings should impact the development of new therapeutics that neutralize chicken IL-1 activity.

## Materials and Methods

### Materials

Ampicillin, imidazole, tris(hydroxymethyl)aminomethane (Tris), sodium chloride, and 2-[4-(2-hydroxyethyl)piperazin-1-yl]ethanesulfonic acid (HEPES) were supplied by USB (Cleveland, OH). Fomblin oil, sodium citrate, lithium sulfate, and ammonium sulfate were purchased from Sigma-Aldrich (St. Louis, MO). Isopropyl β-D-1-thiogalactopyranoside was procured from Protech (Taiwan). *Escherichia coli* BL21 (DE3) was obtained from Yeastern Biotech (Taiwan).

### *In vivo* functional assay for recombinant chicken and human IL-1βs

To evaluate the linearity of the bioactivity of IL-1β *in vivo*, various concentrations of chicken WT IL-1β (1–50 μg/kg body mass) were injected into adult specific-pathogen-free white leghorn chickens (Animal Health Research Institute, Taiwan)^11^. Three independent tests for each concentration of recombinant WT IL-1β on three different chicken samples were performed. After 1 h, the plasma cortisol levels were measured using a Roche E170 Modular immunoassay analyzer (Roche Diagnostics, Mannheim, Germany) as described in^9^. The Institutional Animal Care and Use Committee (IACUC), National Tsing Hua University, Taiwan approved the animal-use protocol (approval number, IACUC:10115). The methods were carried out in accordance with the approved guidelines.

To measure the *in vivo* activities of T7A, R8A, N18A, E25A, H34A, Q36A, R52A, Q64A, Q138A, T117A, E118A, E118K, E118R, Q138A, and WT human and chicken IL-1βs, each proteins was individually injected at a concentration of 10 μg/kg body mass into adult specific-pathogen-free white leghorn chickens. For negative control, phosphate-buffered saline was injected. Independent experiments for each protein that used three chickens were performed. Plasma cortisol levels were measured as described above. Statistical comparison between the mean cortisol levels of each mutant and to that of WT chicken IL-1β was done using student’s t-test^28^.

### CD Spectroscopy

CD spectra between 190 and 260 nm were recorded to estimate the secondary structures of T117A, E118A, E118K, E118R, and WT chicken IL-1β (15 μM) at pH 7.4 in 10 mM potassium phosphate buffer, at 20 °C using an Aviv 202 spectropolarimeter (Aviv Biomedical Inc., NJ)^29^. The average for three CD scans was calculated for each protein, and mean residue ellipticity (deg ∙ cm^2^ ∙ dmol^−1^) was reported for each spectra.

### Surface plasmon resonance

To measure the binding affinities of T117A, E118K, and WT chicken IL-1β for chicken IL-1RI at 25 °C, surface plasmon resonance measurements were acquired with a BIAcore 3000 system (GE Healthcare)^30^. The receptor (5 μg/ml) in 10 mM sodium acetate, pH 5.5 was immobilized on a research-grade CM5 sensor chip through the standard amine-coupling method (final condition, ~370 response units)^31^. Various concentrations of each mutant and WT IL-1β at pH 7.2 in 10 mM HEPES, 150 mM NaCl, 0.001% (v/v) Tween-20 were individually washed at a rate of 30 μl min^−1^ over the flow cell. Both the association and dissociation of binary complexes were allowed to last for 90 s. Data were fit with a 1:1 Langmuir-binding model using the BIAcore 3000 evaluation software BIAevaluation 4.1.

### Cell culture and IL-6 synthesis

The DF-1 chicken fibroblast cells^32^ were seeded into a 6-well plate (Nunc, Rochester, NY) at 1.5 × 10^6^ cells per well and grown until the monolayer was 80–90% confluent. The cells were then washed with phosphate-buffered saline (PBS) and treated with WT, T117A, or E118K IL-1βs at a concentration of 10 ng/ml and the control was treated with Dulbecco’s Modified Eagle’s medium (DMEM). In addition, 0.5 ng/ml of sub-saturating fixed concentration of WT IL-1β mixed with increasing concentration (0.5–40 ng/ml) of the mutants T117A or E118K were also treated with DF-1 cells. After 8 h treatment at 39 °C, the inoculum was removed and the IL-6 expression in DF-1 cell lysates was analyzed using a chicken-specific ELISA kit (Cloud-Clone, Houston, TX)^22^,^23^. The plate was read with the iMark microplate reader (BioRad, Hercules, CA). The IL-6 samples and standards were evaluated in triplicate.

### Crystallization of recombinant chicken IL-1βs

Crystals of mutants T117A, E118A, and E118K (from a solution of concentration 15 mg/ml) were grown at 20 °C using the hanging-drop, vapor-diffusion technique. Hundreds of crystallization conditions were tested using reagents from the following commercial screening kits: Wizard I, II, and III (Emerald BioSystems, WA); Crystal Screen, Crystal Screen 2 and PEG/Ion (Hampton Research, CA); and Clear Strategy Screen I/II (Molecular Dimensions Ltd., Newmarket, UK). Crystals were found to grow the best in the condition of 1.15 M (NH_4_)_2_SO_4_, 0.1 M Tris-HCl, 0.2 M LiSO_4_, pH 8.0 at 20 °C. Each crystal was soaked in fomblin oil before being frozen in liquid nitrogen.

### Structure determination and refinement

X-ray crystallographic data (Supplementary Table 2) were collected at the SPXF beamline BL13B1 of the National Synchrotron Radiation Research Center in Hsinchu, Taiwan, using a mar345 Image Plate Detector (Marresearch GmbH, Germany). The wavelength was 1.0 Å. Diffraction data sets were collected and processed using the HKL-2000 package (HKL Research Inc., VA). The asymmetric unit for each mutant of chicken IL-1β (namely, T117A, E118A, and E118K) contains a single protein molecule in the crystals of orthorhombic space group P2_1_2_1_2_1_. Molecular replacement was performed to generate the mutant structures using the CCP4 program Molrep^33^ with all residues in chicken IL-1β (PDB ID: 2WRY)^11^ as the search model. Data between 30.0 to 3.5 Å and a Patterson radius of 20 Å were used to calculate translation and rotation functions. In the same way, WT chicken IL-1β was used as a template to build the structures of T117A, E118A, and E118K. The initial mutant chicken IL-1β structures were rebuilt and then refined by Coot^34^ and Refmac5^35^, respectively. The refinement statistics are summarized in the Supplementary Table 2. Structure validation was performed by PROCHECK v.3.5.4^21^, and secondary structures were identified using DSSP^36^. The atomic coordinates and structure factors for T117A, E118A, and E118K have been deposited in PDB under the accession codes 4X39, 4X38, and 4X37, respectively.

### Molecular modeling of chicken IL-1RAcP and multiple sequence alignment of chicken IL-1βs

Protein sequences were retrieved from ExPASy Molecular Biology Server (http://www.ebi.expasy.org/). Homology model of the extracellular domain of chicken IL-1RAcP was constructed using the structure of human IL-1RAcP (PDB entry 4DEP) as the template^27^. The two proteins share a 60.9% sequence identity according to ClustalW^37^ (http://www.ebi.ac.uk/clustalw). LigPlot^38^ was used to analyze the atomic interactions between constituent proteins in binary or tertiary (IL-1β/IL-1RI/IL/IL-1RAcP) complexes. PyMOL^39^ was used for molecular visualization and representation of the electrostatic potential surface.

### MD refinement of chicken IL-1β/IL-1RI/IL-1RAcP

MD simulations for the chicken IL-1β/IL-1RI binary complex were reported in^13^. With the human IL-1β/IL-1RI/IL-1RAcP structure (PDB: 4DEP) as the template, we first modeled chicken IL-1RAcP by superimposition onto that of human IL-1RAcP in human IL-1β/IL-1RI/IL-1RAcP and superimposed the last MD snapshot of chicken IL-1β/IL-1RI onto its human equivalent. The resulting chicken ternary complex contained a few steric clashes between pairs of atoms separated by <1 Å at the IL-1β/IL-1RAcP and IL-1RI/IL-1RAcP interfaces To eliminate the steric clashes, we first translated IL-1RAcP away from the ternary complex through a limited number of steps, 1-Å at a time in either of the x, y, or z directions, until no clashes were found. We then pulled all the IL-1RAcP atoms, except for those involved in the steric clashes, back to their original coordinates (before the ‘stepping away’) using targeted MD simulations, with a time-varying force constant reduced from of 5.0 kcal/mol/Å^2^ to pull the aforementioned atoms^40^,^41^,^42^. The procedure brought majority of the IL-1RAcP atoms back to the positions suggested by homology modeling and x-ray crystallography data (per human IL-1RAcP) and result in the closest distance between atoms of IL-1RAcP and the binary IL-1β/IL-1RI complex to be >1.7 Å (compared to 0.23 Å before the treatment). Next, the ternary complex was subjected to energy relaxation and MD-based structural refinement. To do so, the ternary complex was solvated in a water box with dimensions of 128.9 Å × 118.9 Å × 138.5 Å containing nine neutralizing Na^+^ ions. The system was first subjected to energy minimization then heated to 310 K in an NVT ensemble and eventually equilibrated in an isothermal-isobaric ensemble for 10 ns. During the energy minimization, a weak restraint of 5.0 kcal/mol/Å^2^ was applied to restrain the Cα atoms in their original positions, which led to a total Cα rmsd of <1.8 Å between the equilibrated structure and the original ternary structure excluding the atoms involved in original steric clashes. In the MD simulation, the complete electrostatic energy was calculated by Particle Mesh Ewald with a non-bonded distance cutoff of 12 Å. The temperature was maintained at 310 K using the Langevin thermostat with a collision frequency of 2 ps^−1^, and pressure of 1 bar using a Berendsen barostat.

## Additional Information

**How to cite this article**: Chen, W.-T. *et al*. Structure and function of chicken interleukin-1 beta mutants: uncoupling of receptor binding and *in vivo* biological activity. *Sci. Rep.* **6**, 27729; doi: 10.1038/srep27729 (2016).

## Supplementary Material

Supplementary Information
