# Supplementary material for: Structure and function of chicken interleukin-1 beta mutants: uncoupling of receptor binding and in vivo biological activity
Source: Sci Rep. 2016 Jun 9;6:27729. doi: 10.1038/srep27729 (PMC4899739; doi:10.1038/srep27729)
Supplement: Supplementary Information [file srep27729-s1.pdf]

Supplementary File

Structure and function of chicken interleukin-1 beta mutants: uncoupling of receptor binding and *in vivo* biological activity

Wen-Ting Chen[a], Wen-Yang Huang[a], Ting Chen[a], Emmanuel Oluwatobi Salawu[a,b], Dongli Wang[c], Yi-Zong Lee[a], Yuan-Yu Chang[a], Lee-Wei Yang[a,b], Shih-Che Sue[a], Xinquan Wang[c] and Hsien-Sheng Yin[a]*

[a]Institute of Bioinformatics and Structural Biology, and College of Life Sciences, National Tsing Hua University, No. 101, Section 2, Kuang-Fu Road, Hsinchu 30013, Taiwan

[b] Bioinformatics Program, Taiwan International Graduate Program, Academia Sinica, Taipei, 115, Taiwan

[c] School of Life Science, Tsing Hua University, Beijing, China

***Corresponding author:**

Institute of Bioinformatics and Structural Biology, and College of Life Sciences, National Tsing Hua University, No. 101, Section 2, Kuang-Fu Road, Hsinchu 30013, Taiwan

E-mail: hstin@mx.nthu.edu.tw; Tel: +886-3-574-2469; Fax: +886-3-571-5934

Experimental Procedures

Expression and purification of recombinant human and chicken IL-1βs.

The gene for wild-type (WT) chicken interleukin-1 beta (IL-1β) was cloned as described [1] and used as the template to construct the IL-1β mutants. The codons for residues T7, R8, N18, E25, H34, Q36, R52, R54, Q64, T117, E118, and Q138 were replaced with that for alanine using Quikchange site-directed mutagenesis kit reagents (Stratagene, The Netherlands) according to the manufacturer's instructions. The codon for E118 was also replaced with one for lysine or arginine to prepare the mutants E118K and E118R, respectively. Primers used are listed in Supplementary Table 1. The mutated genes were individually cloned into pET-28a(+) (Promega, WI), with an upstream T7 promoter-His_6 tag, and expressed in *Escherichia coli* BL21 (DE3). The gene sequences were confirmed by DNA sequencing (Mission Biotechnology Inc., Taiwan).

The cells were cultured in Luria-Bertani medium containing 50 mg/ml ampicillin. When the OD_{600} = 0.6, isopropyl-thio-β-D-galactopyranoside (0.4 mM final concentration) was added into each culture to induce protein expression [2]. Expressed protein was purified as described [3]. Briefly, whole cells were lysed by sonication in 25 mM Tris-HCl, 100 mM NaCl, pH 7.4, then centrifuged at 100,000 × *g* for 30 min at 4°C. The His-tagged proteins were purified by Co^{2+}-affinity column chromatography

(BD Biosciences, CA). The column was washed with 100 mM imidazole, 25 mM Tris-HCl, 100 mM NaCl, pH 7.4, and protein was eluted in the same solution but containing 300 mM imidazole. A Centricon YM-10 centrifugal filter device (Millipore, MA) was then used to remove imidazole and to concentrate each protein. The final protein products were characterized by SDS-PAGE [4] and peptide mass fingerprints were obtained using an Autoflex III MALDI-TOF Mass Spectrometry (Bruker Daltonics Inc., Billerica, MA) [5]. Briefly, the Sophisticated Numerical Annotation Procedure (SNAP) algorithm was used for spectrum annotation using the following detailed metrics: peak detection algorithm, SNAP; signal-to-noise threshold, 25; relative intensity threshold, 0%; minimum-intensity threshold, 0; maximal number of peaks, 50; quality factor threshold, 1000; SNAP average composition, averaging; baseline subtraction, median flatness, 0.8; median level, 0.5. The spectrometer was adjusted with a peptide adjustment standard (Bruker Daltonics), and internal adjustment was carried out using trypsin autolysis peaks at m/z 842.51 and 2211.10. Peaks in the mass range of m/z 800 to 3000 were used to achieve a peptide mass fingerprint that was searched against the updated Swiss-Prot/TrEMBL database. Protein concentrations were determined using Bio-Rad Quick Start Bradford Protein Assay reagents (Bio-Rad, CA) with bovine serum albumin as the standard. Mutant IL-1βs (15 mg/ml) in 25 mM Tris-HCl, 100 mM NaCl, pH 7.4 served as stock

solutions.

Protein docking of the IL-1R/IL-1β complex onto IL-1RAcP

Geometric-hashing-accelerated PatchDock [6] was used to dock IL-1R/IL-1β complex, obtained from the last snapshot of the MD simulation, onto homology modeled chicken IL-1RAcP, in order to cross validate the adequacy of the ternary chicken complex (IL-1R/IL-1β/IL-1RAcP) modeled from its human equivalent. The highest scored 200 docked poses were evaluated and visually inspected. The pose with the most favorable energy and the lowest rmsd from the human IL-1RI/IL-1β complex was selected as the complex model for each chicken mutant.

Molecular dynamics simulations of T117A, E118A, and E118K complexed to IL-1RI

Mutant IL-1βs and chicken IL-1RI in both the free and complexed forms are simulated by Gromacs 5.0 package (www.gromacs.org). Hydrogen atoms were added using the GROMOS96 45a3 force field [7]. Water molecules in each crystal structure were retained. Free and complexed starting structures were individually immersed into a rectangular box containing pre-equilibrated, single-point charge water models with 12 Å as the shortest distance between the box edge and the solutes, resulting in ~15,500 water molecules in the simulation box for all runs. To achieve electro-neutrality, single-point charge water molecules were randomly replaced with

55 sodium ions for the IL-1RI and IL-1RI/mutant IL-1β simulations and with 49 chloride ions for each free mutant run. The MD simulations began with a steepest descent energy minimization for 100 ps to relax any steric clashes in the initial systems. Subsequently, each simulation was performed using decreasing positional restraints to further equilibrate the systems. After equilibration, all systems were simulated for 6 ns as an isobaric-isothermal ensemble using periodic boundary conditions. The temperature was maintained by coupling the protein, solvent, and counter-ions to a 300-K temperature bath with a coupling constant τT of 0.1 ps. The pressure was controlled using Berendsen's weak-coupling algorithm set at 1 atm with a coupling constant τP of 2 ps. Lennard-Jones interactions were truncated at a cut-off distance of 12 Å. Long-range electrostatic interactions were modeled using the particle mesh Ewald algorithm. The linear constraint solver algorithm was used to restrain all bond lengths. The integration time step was 2 fs, and the coordinates of individual systems were collected every 0.5 ps during the production runs.

References

1 Cheng, C. S. *et al.* Structural and functional comparison of cytokine interleukin-1 beta from chicken and human. *Mol Immunol* **48**, 947-955 (2011).

2 Cheng, C. S. *et al.* Crystal structure and biophysical characterisation of Helicobacter pylori phosphopantetheine adenylyltransferase. *Biochem Biophys Res Commun* **408**, 356-361 (2011).

3 Chen, W. T. *et al.* Circular permutation of chicken interleukin-1 beta enhances its thermostability. *Chem Commun (Camb)* **50**, 4248-4250 (2014).

4 Laemmli, U. K. Cleavage of structural proteins during the assembly of the

head of bacteriophage T4. *Nature* **227**, 680-685 (1970).
5 Chen, W. T. *et al.* Proteomics analysis of the DF-1 chicken fibroblasts infected with avian reovirus strain S1133. *PLoS One* **9**, e92154 (2014).
6 Schneidman-Duhovny, D., Inbar, Y., Nussinov, R. & Wolfson, H. J. PatchDock and SymmDock: servers for rigid and symmetric docking. *Nucleic Acids Res* **33**, W363-367 (2005).
7 Schuler, L. D., Walde, P., Luisi, P. L. & van Gunsteren, W. F. Molecular dynamics simulation of n-dodecyl phosphate aggregate structures. *Eur Biophys J* **30**, 330-343 (2001).

Supplementary Table 1. Primers used in this study

Mutant	Forward Primer (5' → 3')	Reverse Primer (5' → 3')
T7A	CCCGCCTTCCGCTACGCCCGCTCACAG	GAAGGACTGTGAGCGGGCGTAGCGGAAG
R8A	GCCTTCCGCTACACCGCCTCACAGTCCTTC	GTCGAAGGACTGTGAGGCGGTGTAGCGGG
N18A	CTTCGACATCTTCGACATCGCCCAGAAGTGC	CAGCACGAAGCACTTCTGGGCGATGTCGAAG
E25A	GAAGTGCTTCGTGCTGGCGTCACCCACC	GCTGGGTGGGTGACGCCAGCACGAAG
H34A	CAGCTGGTGGCCCTGGCCCTCCAGGGG	GGAGGGCCCCTGGAGGGCCAGGGCCACCAG
Q36A	GTGGCCCTGCACCTCGCGGGGCCCTCC	GCTGGAGGAGGGCCCCGCGAGGTGCAG
R52A	CTCAACATTGCGCTGTACGCGCCCCGAGGC	GCCCCGTGGGCCTCGGGGCGCGTACAGCGC
R54A	GCGCTGTACCGGCCCGCAGGCCCACGG	GCTGCCCCGTGGGCCTGCGGGCCGGTACAG
Q64A	GGCAGCGCTGGAACTGGGGCGATGCCAGTG	CAGTGCCACTGGCATCGCCCCAGTTCCAGC
T117A	CGCCTGGACAGCCCGGCTGAGGGCAC	CGTGGTGCCCTCAGCCGGGCTGTCC
E118A	GGACAGCCCGACTGCGGGCACCACG	GCGCGTGGTGCCCGCAGTCGGGCTG
E118K	CTGGACAGCCCGACTAAGGGCACCACGC	GAAGCGCGTGGTGCCCTTAGTCGGGCTG
E118R	CTGGACAGCCCGACTCGGGGCACCACGC	GAAGCGCGTGGTGCCCCGAGTCGGGCTG
Q138A	CATCTGCACCTCCCTGGCGCCCCGGCAG	CACGGGCTGCCGGGGCGCCAGGGAGGTG

Supplementary Table 2. Data collection and refinement statistics

T117A	
**Data Collection**	
Wavelength (Å)	1.0000
Temperature (K)	100
Resolution (Å)	1.60 (1.66–1.60)[a]
Space group	$P2_12_12_1$
Unit cell dimensions	
a, b, c (Å)	35.61, 38.50, 102.04
α, β, γ (°)	90.0, 90.0, 90.0
No. of unique reflections	19,057
No. of observations	128,625
Redundancy	6.8 (6.5)[a]
Completeness (%)	99.7 (99.9)[a]
Mean I/σ (I)	39.14 (6.93)[a]
Protein molecules	1
Matthew coefficient ($Å^3$ Da^{-1})	1.85
Solvent content (%)	33.64
Wilson *B*-factor ($Å^2$)	21.3
R_{merge}	0.043 (0.251)[a]
**Refinement**	
Resolution range (Å)	26.16–1.61
No. of reflections used	17,773
No. of protein atoms	1,252
No. of water molecules	47
Average *B*-factors ($Å^2$)	
All protein atoms	15.187
Waters	17.184
R_{work}/R_{free} (%)	20.05/23.66
Ramachandran plot statistics (%)	
Residues in most favored regions	90.1
Residues in additional allowed regions	9.9
Residues in generously allowed regions	0
Residues in disallowed regions	0
Rmsd bond length (Å)	0.015
Rmsd bond angle (°)	1.762
PDB entry	4X39

[a]Outer shell value.

Supplementary Table 2. Data collection and refinement statistics (continued)

E118A	
**Data Collection**	
Wavelength (Å)	1.0000
Temperature (K)	100
Resolution (Å)	2.12 (2.18–2.12)[a]
Space group	$P2_12_12_1$
Unit cell dimensions	
a, b, c (Å)	35.67, 35.76, 102.27
α, β, γ (°)	90.0, 90.0, 90.0
No. of unique reflections	7,853
No. of observations	37,880
Redundancy	4.9 (4.8)[a]
Completeness (%)	98.1 (99.9)[a]
Mean I/σ (I)	13.60 (2.90)[a]
Protein molecules	1
Matthew coefficient ($Å^3$ Da^{-1})	1.94
Solvent content (%)	36.03
Wilson *B*-factor ($Å^2$)	33.1
R_{merge}	0.112 (0.582)[a]
**Refinement**	
Resolution range (Å)	24.5–2.12
No. of reflections used	6,564
No. of protein atoms	1,202
No. of water molecules	27
Average *B*-factors ($Å^2$)	
All protein atoms	26.410
Waters	24.109
R_{work}/R_{free} (%)	18.98/24.72
Ramachandran plot statistics (%)	
Residues in most favored regions	89.8
Residues in additional allowed regions	10.2
Residues in generously allowed regions	0
Residues in disallowed regions	0
Rmsd bond length (Å)	0.010
Rmsd bond angle (°)	1.456
PDB entry	4X38

[a]Outer shell value.

Supplementary Table 2. Data collection and refinement statistics (continued)

E118K	
**Data Collection**	
Wavelength (Å)	1.0000
Temperature (K)	100
Resolution (Å)	1.63 (1.69–1.63)[a]
Space group	$P2_12_12_1$
Unit cell dimensions	
a, b, c (Å)	35.58, 102.50, 37.82
α, β, γ (°)	90.0, 90.0, 90.0
No. of unique reflections	17,881
No. of observations	154,707
Redundancy	8.8 (7.9)[a]
Completeness (%)	98.7 (95.6)[a]
Mean I/σ (I)	66.87 (14.23)[a]
Protein molecules	1
Matthew coefficient ($Å^3\ Da^{-1}$)	1.82
Solvent content (%)	32.56
Wilson *B*-factor ($Å^2$)	27.0
R_{merge}	0.054 (0.137)[a]
**Refinement**	
Resolution range (Å)	29.2–1.63
No. of reflections used	16,734
No. of protein atoms	1,237
No. of water molecules	43
Average *B*-factors ($Å^2$)	
All protein atoms	25.554
Waters	23.586
R_{work}/R_{free} (%)	19.76/22.89
Ramachandran plot statistics (%)	
Residues in most favored regions	98.1
Residues in additional allowed regions	1.9
Residues in generously allowed regions	0
Residues in disallowed regions	0
Rmsd bond length (Å)	0.025
Rmsd bond angle (°)	2.457
PDB entry	4X37

[a]Outer shell value.

Supplementary figure legends

Supplementary Fig. 1.

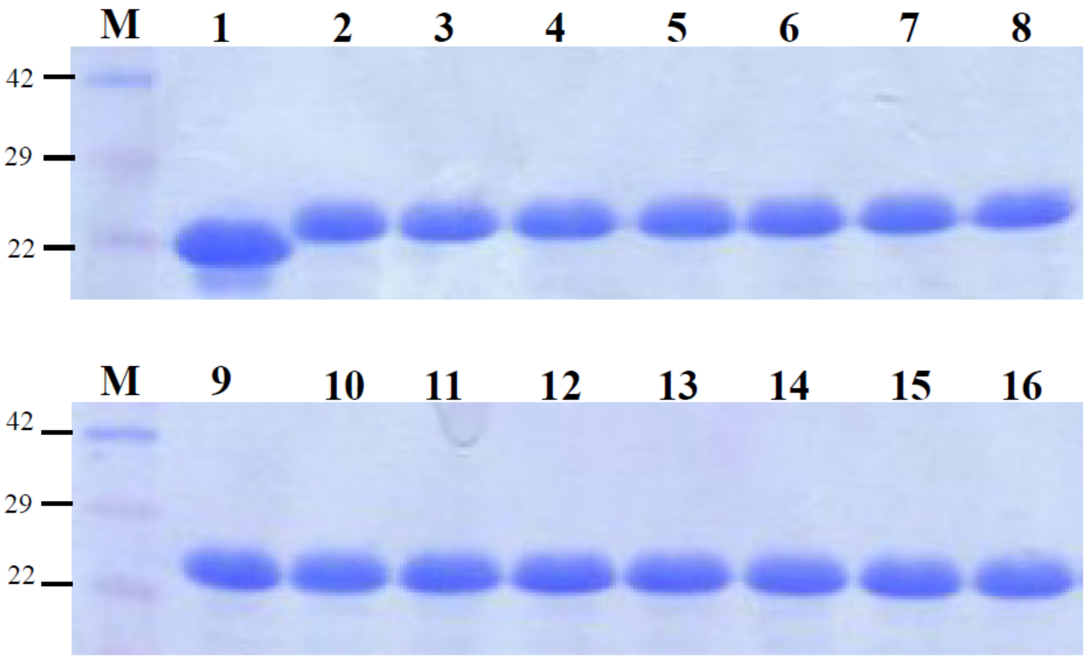

Supplementary Fig. 1. SDS-PAGE (15% (w/v) acrylamide gels) by Coomassie blue staining of purified recombinant WT IL-1β and its mutants. All lanes were loaded with 4 μg of protein. The molecular masses of the protein standards (kDa, lane M) are shown to the left of the gel. Lanes 1: WT human IL-1β; 2: WT chicken IL-1β; 3: T7A; 4: R8A; 5: N18A; 6: E25A; 7: H34A; 8: Q36A; 9: R52A; 10: R54A; 11: Q64A; 12: T117A; 13: E118A; 14: E118K; 15: E118R; 16: Q138A.

Supplementary Fig. 2.

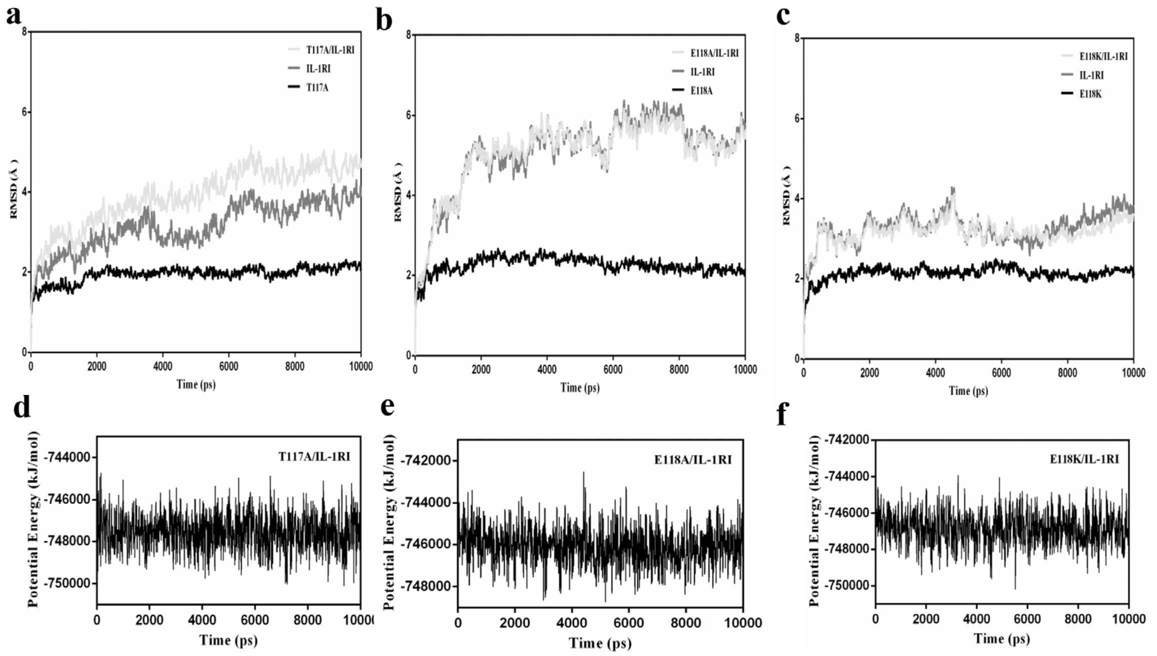

Supplementary Fig. 2. MD trajectories for free T117A, E118A, E118K, and IL-1RI and for the binary mutant/IL-1RI complexes. (a) Averaged rmsd values for the heavy atoms of T117A, IL-1RI, and T117A/IL-1RI. (b) Averaged rmsd values for the heavy atoms of E118A, IL-1RI, and E118A/IL-1RI. (c) Averaged rmsd values for the heavy atoms of E118K, IL-1RI, and E118K/IL-1RI. For panels a-c, the light gray line, gray line, and black line represent the complex, free IL-1RI, and free mutant trajectories, respectively. (d-f) From left to right, potential energies for trajectories of the T117, E118, and E118K complexes.

Supplementary Fig. 3.

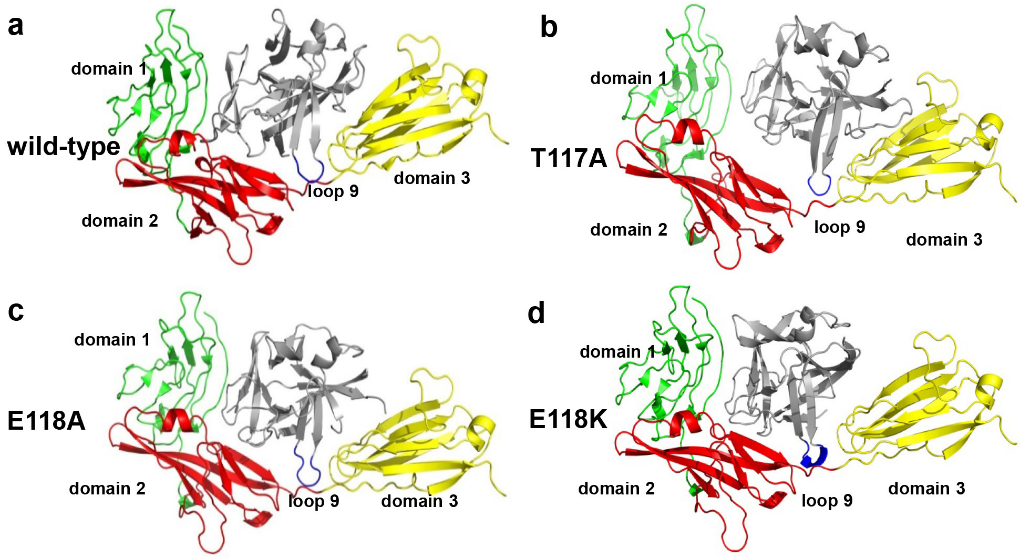

Supplementary Fig. 3. Ribbon diagrams of the binary IL-1RI/WT and mutant IL-1β complexes. (a) WT IL-1β, (b) T117A, (c) E118A, and (d) E118K WT IL-1β, T117A, E118A, and E118K are colored gray with loop 9 colored blue. IL-1RI domains 1, 2, and 3 are colored green, red, and yellow, respectively.
